# Neuroprotective Mechanisms of Taurine against Ischemic Stroke

**DOI:** 10.3390/brainsci3020877

**Published:** 2013-06-03

**Authors:** Janet Menzie, Howard Prentice, Jang-Yen Wu

**Affiliations:** 1 Program in Integrative Biology, Florida Atlantic University, Boca Raton, FL 33431, USA; E-Mail: jmenzie@fau.edu; 2 Department of Biomedical Sciences, Florida Atlantic University, Boca Raton, FL 33431, USA

**Keywords:** ischemic stroke, taurine, neuroprotective mechanisms, glutamate excitotoxicity, mitochondrial dysfunction, endoplasmic reticulum stress, oxidative stress, inflammation, clinical trial

## Abstract

Ischemic stroke exhibits a multiplicity of pathophysiological mechanisms. To address the diverse pathophysiological mechanisms observed in ischemic stroke investigators seek to find therapeutic strategies that are multifaceted in their action by either investigating multipotential compounds or by using a combination of compounds. Taurine, an endogenous amino acid, exhibits a plethora of physiological functions. It exhibits antioxidative properties, stabilizes membrane, functions as an osmoregulator, modulates ionic movements, reduces the level of pro-inflammators, regulates intracellular calcium concentration; all of which contributes to its neuroprotective effect. Data are accumulating that show the neuroprotective mechanisms of taurine against stroke pathophysiology. In this review, we describe the neuroprotective mechanisms employed by taurine against ischemic stroke and its use in clinical trial for ischemic stroke.

## 1. Introduction

Stroke is one of the world’s leading causes of death and disability [1,2]. The World Health Organization (WHO) reported that 5.71 million people died of stroke in 2004 with an estimated acceleration to 7.8 million by 2030 [3]. In keeping with this estimated trajectory of stroke, it was also reported that within the next decade there will be a 12% global rise in stroke morbidity [4]. The two types of stroke are ischemic stroke and hemorrhagic stroke. Of the two approximately 85% are ischemic [5]. 

Ischemic stroke (cerebral ischemia) is due to a partial or complete reduction in blood flow to the brain. The ischemia may be global due to cardiac arrest or focal due to a blockage in a specific blood vessel. Brain regions most susceptible to damage are hippocampal CA1 and neocortical layers 3, 5, and 6 [6]. Cerebral hypoxia (a reduction of cerebral oxygen) generally accompanies an ischemic insult but may also occur without the loss of blood flow as in the case of respiratory arrest, near-drowning or carbon monoxide poisoning [7]. Insufficient oxygen and glucose supply in cerebral ischemia leads to unsustainable cellular homeostasis which initiates cell injury. Cellular injury progresses as a result of excitotoxicity, ionic imbalance, oxidative and nitrosative stresses, endoplasmic reticulum (ER) stress and mitochondrial disturbances, ultimately resulting in programmed cell death and necrosis [8]. Pathologically the ischemic infarct is observed as a central core, a region where cells undergo anoxic depolarization and never repolarize. Cells in the core eventually become necrotic [9] immediately surrounding the core (perifocal region) is the ischemic penumbra, a region where cells receive some perfusion via collateral circulation and may repolarize but they are still highly vulnerable to injury [9]. Cells in the penumbra are subject to apoptosis but may be rescued by neuroprotective measures [10]. If the ischemic process is not arrested the ischemic core will recruit the perifocal penumbra by a process called “spreading depression”; which is unarrested massive depolarization [11,12]. Manifestation of cerebral ischemia involves neurological deficit in cognition, motor and sensory functions, the severity of which reflects the location and size of the damaged area. 

Due to the multiple pathophysiological mechanisms observed in ischemic stroke/cerebral ischemia, current treatments remain mostly ineffective apart from thrombolytic therapy which uses thrombolytic recombinant tissue plasminogen activators (rt-PA) [13] such as alteplase [14]. This therapy allows only a 3–4.5 h window for effective treatment. It therefore becomes critical to develop other compounds that are multipotential in addressing the diverse pathological mechanisms in ischemic stroke/cerebral ischemia. 

**Figure 1:**
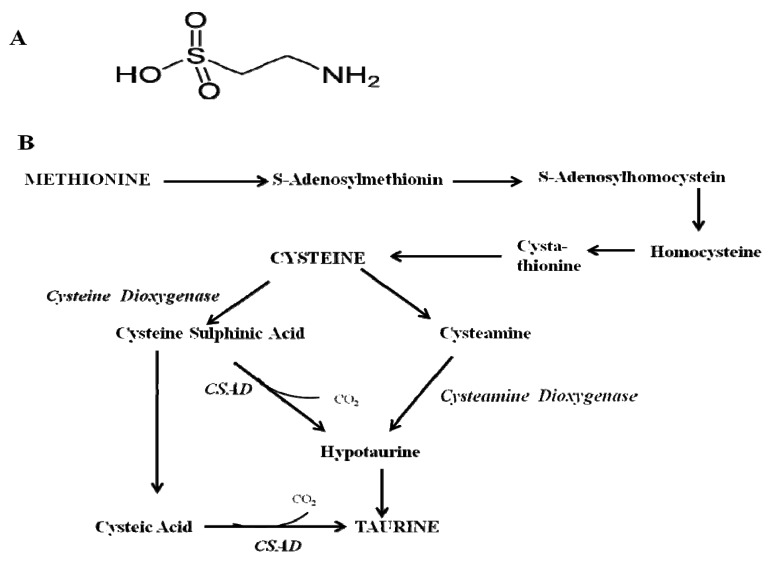
The structure and biosynthesis of taurine. (**A**) Chemical structure of taurine. (**B**) Major biochemical pathways of taurine biosynthesis. Methionine is converted to cysteine via transulfuration. The enzyme cysteine dioxygenase oxidizes cysteine to cysteine sulphinic acid which is further decarboxylized to form hypotaurine by the enzyme, cysteine sulphinic acid decarboxylase (CSAD). Cysteine may also be converted to panteetheine via coenzyme A and then to cysteamine, which is oxidized to form hypotaurine. The oxidation of hypotaurine results in taurine as the end product. Cysteine sulphinic acid may also be converted to cysteic acid which is then decarboxylated to form taurine.

Taurine (2-amino-ethanesulfonic acid) is a sulphur containing, free amino acid, (Figure 1A) that is abundantly found in mammals [15]. Unlike other amino acids the presence of a sulphur group instead of a carboxyl group prevents it from being incorporated into proteins. It is mostly found in excitable tissues such as the brain, retina, cardiac muscle and skeletal muscle [16,17,18,19,20,21,22] and it is synthesized by methionine and cysteine metabolism with cysteine sulphinic acid decarboxylase (CSAD) being the rate-limiting enzyme [23,24] (Figure 1B). Apart from being synthesized endogenously, taurine may also be obtained from meat, dairy products, poultry, fish and shellfish [25,26]. While it was first discovered as a component of ox (*Bos tauru*; from which its name is derived) bile in 1827, it had taken over a century before insights into its physiological functions were made. Early concepts of its physiological functions were provided in a study by Curtis and Watkins [27]. They demonstrated that taurine could be a neurotransmitter which was later supported by Davison and Kaczmarck [28]. There is growing evidence of its physiological importance. Hayes and colleagues [29] reported that cats fed a taurine deficient diet developed central retinal degeneration. This report by Hayes and colleagues emphasized that taurine was essential in species that are unable to synthesize it, such as cats. The importance of taurine in retinal function was also supported by several authors [30,31,32]. Taurine is also a key player in: cardiovascular and muscular skeletal functions [33,34,35], regulating the release of pancreatic insulin [36] and renal function [26]. Interestingly, taurine’s role in brain development involves the differentiation and migration of cerebellar, pyramidal and visual cortical cells in cats and monkeys [37,38,39,40] as well as playing a role in both embryonic and adult neurogenesis [40,41]. These lines of evidence highlight a few of its physiological functions, for review [19,42,43]. Taurine also exhibits its potential to be effective against many diseases as observed in animal models of Type 1 and Type 2 diabetes [44,45,46], atherosclerosis [47] and neurological disorders such as Alzheimer’s, Parkinson’s and Huntington’s diseases [48]. Its depletion has also been reported in cardiomyopathy [49,50]. 

### Physiological Function of Taurine in Central Nervous System

Taurine is able to cross the blood-brain barrier [51,52] and displays a plethora of functions in the central nervous system (CNS) [42,53,54]. In the CNS it plays a role in: neuromodulation [55,56,57], osmoregulation [33,58], the maintenance of calcium homeostasis [59,60,61,62,63], membrane stabilization [64], anti-oxidation [65,66], anti-inflammation [67,68] and neuroprotection [59,69,70,71,72,73] and is also seen as a trophic factor during CNS development [40,74]. Its neuroprotective effect is observed against l-glutamate induced excitotoxicity whereby it counteracts the glutamate-induced increase of intracellular calcium through L-, P/Q-, N-type voltage-gated calcium channels (VGCCs) and the *N*-methyl-d-aspartate (NMDA) receptor, thus preventing glutamate-induce membrane depolarization [59,69]. 

Although taurine is not definitively classified as a neurotransmitter it fulfills most of the necessary criteria: it is associated with synaptic membrane structures [75] and both taurine and its synthesizing enzyme CSAD, are co-localized in presynaptic neuronal terminals [23,76,77]; stimulated-taurine release is both calcium dependent (at a potassium concentration up to 40mM) and calcium independent (at a potassium concentration greater than 40mM) [78]; taurine is taken up into the cell by a sodium-dependent taurine transporter (TauT) [79,80,81]. It modulates neurotransmission by eliciting inhibitory neuronal transmission through GABA_A _receptors, glycine receptors and putative taurine receptors [82,83,84,85]. The only criterion that has not been met for it to be fully classified as a neurotransmitter is the cloning of a taurine specific receptor. Previously we identified a putative taurine receptor [86]; our study and the later investigation by Frosini *et al.* 2003 [87] demonstrated that the receptor is neither activated nor antagonized by structurally similar amino acids such as glutamate, gamma-amino butyric acid (GABA) and glycine.

The fundamental pathophysiological mechanisms involved in ischemic stroke are glutamate excitotoxicity, calcium imbalance and oxidative stress which individually or collectively results in cell death. Therefore taurine’s role as an inducer of inhibitory neurotransmission, an anti-oxidant, neuromodulator, regulator of calcium homeostasis and neuroprotector, potentially makes it an ideal therapeutic agent for ischemic stroke. This review will focus on previous and current studies of taurine’s neuroprotective effect on ischemic stroke with an insight to the underlying mechanisms employed against the pathophysiology of ischemic stroke and the possibility of its use in clinical trials for ischemic stroke patients. 

## 2. Neurochemical Mechanisms of Ischemic Stroke

Three major mechanisms attribute to brain damage in ischemic stroke; glutamate excitotoxicity which leads to an increase of intracellular cytosolic calcium, acidosis and increased production of free radicals. Within 10–20 s of the insult there is a loss of consciousness and loss of neuronal electrical activity within the ischemic area [88,89]. This initial 20 s is followed by the failure of energy-dependent pumps, such as the Na^+^/K^+^-ATPase and Ca^2+^-ATPase pump, impairment of the energetics required to maintain ionic gradients, and a resulting imbalance of ion homeostasis [90]. Increased influx of Na^+^ and reduced efflux of K^+^ induce membrane depolarization of neurons and glia, is followed by the resulting influx of Ca^2+^ through VGCC’s [91] and release of the excitatory amino acid; glutamate [92]. Both *in vitro* and *in vivo* studies have shown a massive release of glutamate during ischemic stroke [93,94,95,96,97,98]. Interestingly, glutamate release during ischemic stroke can also be Ca^2+^-independent, distinct from exocytosis, by passing through volume-regulated anion channels (VRAC) [99,100,101]. Another source that accounts for the increased accumulation of extracellular glutamate is the reversal of the glutamate transporter (GLT-1) which occurs due to increases in intracellular Na^+^ and extracellular K^+^ [102,103]. 

Contributions to cytosolic Ca^2+^ overload in the ischemic cell arise from many processes. Initial increases occur through the action of VGCCs and the reverse mode of the Na**^+^**/Ca^2**+**^ exchanger [104]. Excessive extracellular glutamate hyperactivates ionotropic and metabotropic glutamate receptors, NMDA, AMPA/Kainate and mGluRs respectively [105]. Hyperactivation of these receptors results in augmented Ca^2**+**^ permeability of the receptors especially through the NMDA receptors. Normally AMPA/Kainate receptors are not permeable to Ca^2**+**^ but ischemia activates a population of AMPA receptors that are Ca^2+^ permeable [106]. Activation of mGluRs results in Ca^2**+**^ release from calcium stores, such as the endoplasmic reticulum (ER) via the binding of inositol, 1,4,5-triphosphate (IP**_3_**) to its receptor (IP_3_R). The ryanodine receptor (RyR), located on the ER membrane will also release Ca^2**+**^ from the ER via a calcium-induced-calcium-release mechanism [107,108]. An imbalance in ER calcium homeostasis propagates ER stress and resultant apoptosis [109]. Extracellular and intracellular acidosis (fall from pH 7.3 to 6.2) develop concurrently with the imbalance of calcium homeostasis in response to the production of lactic acid during anaerobic metabolism. Acidosis can specifically elicit early necrosis and delay apoptosis in the ischemic cell [110]. Although acidosis may be a direct cause of cell damage/death, it also augments increased cytosolic calcium through acid-sensing ion channels (ASICs) [111]. 

Excessive cytosolic calcium initiates cellular events, by activating catabolic enzymes such as proteases [112], phospholipases and endonucleases [113], which initiate the development of cellular injury and cell death. For example increases in [Ca^2^**^+^**]_i_ activate phospholipase A2 (PLA_2_) which acts on membrane phospholipids, altering membrane structure and rendering it more permeable [114]. One important physiological function of the mitochondria is to sequester cytosolic calcium. The mitochondrion becomes dysfunctional in brain ischemia due to excessive intra-mitochondrial calcium, eliciting excessive production of free radicals/reactive oxygen species (ROS) such as superoxide, hydrogen peroxide and nitric oxide [115,116,117]. The production of ROS is augmented in the reperfusion phase of brain ischemia due to the reintroduction of oxygen to the injured site. Unscavenged excessive ROS causes protein- and lipid-oxidation, interfering with membrane structure and causing DNA damage which inevitably leads to necrotic and apoptotic cell death [118,119]. The pathogenesis of ischemic stroke also involves a deficiency of inhibitory GABAergic mechanisms due to the activation of the mGlu1 receptor located on GABAergic neurons. Extracellular glutamate activation of presynaptic mGlu1 receptors on GABAergic interneuron suppress the release of GABA [120] resulting in a subsequent imbalance between the excitatory and inhibitory neurotransmitter systems. 

## 3. Anti-Ischemic Stroke Mechanisms of Taurine

A 90% reduction in stroke incidence was observed in a genetic rat model of stroke (SHRSP: Stroke-prone spontaneously hypertensive rat), fed a fish diet rich in taurine [121]. We have demonstrated that taurine neurons in rat hippocampus are relatively inert to cerebral ischemia [122]. These observations called attention to the prospect that the prevention and/or amelioration of ischemia in these reports could be that the onset of an ischemic insult triggered the protective mechanism(s) of taurine, thereby protecting the brain from the ischemic on slate [123]. In a rabbit model of transient focal ischemia (trans-orbital three vessel occlusion), using *in vivo* microdialysis, Matsumoto and colleagues measured the evoked release of excitatory (glutamate and aspartate) and inhibitory (GABA and taurine) amino acids into the extracellular space of the cerebral cortex. The study showed a concomitant increase of extracellular glutamate, aspartate and GABA as well as taurine [124]. The reduction of glutamate and taurine immunolabeling of pyramidal cell bodies provide an anatomical support to Matsumoto’s study of an efflux of these amino acids from neurons [125]. Several lines of evidence from both *in vitro* and *in vivo* experiments have provided additional support for the increased release of taurine in cerebral ischemia [126,127,128,129,130]. Recently it has been shown that the ischemic-induced release of taurine is glutamate receptor-mediated [131]. Saransaari and Oja demonstrated that brain stem slices of the adult mouse released preloaded [H^3^] taurine under ischemic conditions. The release was enhanced in the presence of both ionotropic (NMDA, AMPA and kainate) and metabotropic (mGluR I and mGluR III) glutamate receptors agonists while it was reduced in the presence of their antagonists. This indicates that in ischemia, the activation of glutamate receptors will enhance the consequential release of taurine. This increase in extracellular taurine may serve as compensatory protective response which aims at counteracting glutamate excitotoxicity.

### 3.1. Taurine’s Neuroprotective Effect against Glutamate Excitotoxicity

We and other investigators have established that taurine has a protective effect in cultured neurons against glutamate-induced excitotoxicity [59,61,69,132,133,134,135,136,137]. Such observations from these studies have shown that taurine’s neuroprotective mechanism acts through the maintenance of intracellular calcium homeostasis via the inhibition of the Na^+^/Ca^2+ ^exchanger reverse mode [59], inhibition of L-, P/Q-, N-type voltage-gated calcium channels [69], prevention of Ca^2+ ^influx through NMDA receptor calcium channels [138], inhibition of calcium release from the endoplasmic reticulum [139], and the maintenance of intra-mitochondrial calcium homeostasis [63]. Although the reported neuroprotective mechanisms of taurine were observed in glutamate-induce neuronal damage using a cell culture system there is also evidence that taurine’s neuroprotective effects are observed in both *in vitro* and *in vivo* models of ischemic stroke. Schurr and colleagues [140] reported that hypoxia-induced hippocampal brain slices pre-treated with taurine improves the synaptic function of hippocampal neurons in a dose-dependent manner by attenuating Ca^2+ ^movement across the membrane. Ricci and colleagues reported that taurine protects rat brain cortical slices against oxygen/glucose deprivation-reoxygenation [141]. In a chemically-induced (2,4-dinitrophenol) hypoxic model of rat hippocampal neuronal cultures, intracellular calcium was inhibited by taurine (3mM) [142]. Taurine also protects against glutamate excitotoxicity via the activation of GABA_A_ and strychnine-sensitive glycine receptors. This was shown in a rat model of transient focal ischemia, whereby the middle cerebral artery (MCA) was occluded for 2 h [143]. In this study taurine was given pre- and post-ischemia and in several paradigms taurine significantly reduced the neurological deficit score (a score given to indicate the severity of ischemia based on the animal’s behavior), infarct volume and brain water content compared to control animals. It was also noted that this effect of taurine was only partially reduced with either the GABA_A_ antagonist (bicuculline) or the glycine antagonist (strychnine) but completely abolished with the co-application of both antagonists, indicating that taurine’s effect is through both receptors. These studies on hypoxic-ischemic stroke models add credence to taurine’s neuroprotective mechanism of eliciting inhibitory neurotransmission, thereby attenuating the deplorizing-evoked component of glutamate excitotoxicity [144], maintaining calcium homeostasis as mentioned above. The reduction of intracellular Ca^2**+**^ overload has resulting anti-necrotic and anti-apoptotic effects because of the consequential influence on the regulation of activities of calpains and caspases and on mitochondrial function.

### 3.2. Taurine’s Neuroprotective Effect on Mitochondrial Dysfunction, Calpain and Caspase Activities

Excessive intracellular Ca^2+^ results in mitochondrial calcium overload and mitochondrial dysfunction [145]. Dysfunctional mitochondrial related-events involves; the collapse of the mitochondrial membrane potential (Δψm), an opened mitochondrial permeability transition pore (MPTP: a pore that spans both the inner and outer mitochondrial membrane), the subsequent release of pro-apoptotic proteins, such as cytochrome C [146,147,148] and the uncoupling of oxidative phosphorylation with a consequential decrease in adenosine tri-phosphate (ATP) production [149]. Interestingly, taurine is found in high concentration in the mitochondria [150,152] and evidence has been presented showing taurine to be a buffering agent for intra-mitochondrial calcium level [62,153] as well as buffering the pH of the mitochondrial matrix [154]. The buffering action of taurine in the mitochondria has proven to be a protective mechanism in ischemic stroke pathology [64]. El Idrissi demonstrated that taurine was able to maintain intra-mitochondrial calcium homeostasis in glutamate-induced excitotoxicity in cerebellar granule cells exposed to increase [Ca^2+^]_i_ [63]. Using a rat retinal ganglion cell line exposed to hypoxia for 24 hrs, Chen and colleagues [155] demonstrated that taurine prevented mitochondrial dysfunction. The investigators showed a reduction in the extent to which the MPTP was open in taurine-treated cells. The decrease in the Δψm was more significant in non-taurine treated cells than the treated cells and that there was an overall increase in ATP production, again in the taurine-treated cells [155]. Although Chen and colleagues did not report on the precise mechanism of taurine’s protective action against mitochondrial dysfunction, it can be postulated this is due to taurine’s capacity to either buffer intra-mitochondrial calcium [63] and/or buffer the alkaline pH of the mitochondria’s matrix [154,156]. Intriguingly, as part of a post-transcriptional modification, taurine is incorporated into uridine of the mitochondria’s transfer RNA^Lue(UUR)^ [mt tRNA^Lue(UUR)^] at the wobble anticodon position, thus modifying uridine into 5-taurinomethyluridine [157]. A mutated mt tRNA^Lue(UUR)^ hinders the incorporation of taurine, resulting in a defective mt tRNA^Lue(UUR)^ that is unable to recognize it cognate codon, UUG [158]. This defect causes mitochondrial dysfunction which appears as stroke-like episodes (one of the conditions associated with MELAS: mitochondrial myopathy, encephalopathy, lactic acidosis and stroke-like episodes) [158,159]. Rikimaru and colleagues using a culture system of MELAS patient-derived pathogenic cells reported that a high concentration of taurine (40 mM for 4 day exposure) was able to reverse the mitochondrial dysfunction observed in these patients. In their study, taurine increase the oxygen consumption rate, increased the Δψm in a time-dependent and dose-dependent (0 mM, 20 mM and 60 mM) manner and reduced oxidative stress [160]. The study also reported that the MRI of MELAS patients, treated with taurine, showed a reduction in the spread of the ischemic infarct to different brain region. These protective mechanisms of taurine in these patients were evident as an amelioration of stroke-like episodes. 

The activation of calpains and caspases results in apoptotic and necrotic cell death. There is increasing evidence that both calpains and caspases play a major role in ischemia-mediated cell death [161,162,163,164,165,166,167]. Calpains (µ-calpain and m-calpain) are members of the cysteine protease family, are activated by Ca^2**+**^ (micromolar [Ca^2**+**^]_i_ and millimolar [Ca^2**+**^]_i_, respectively) and are endogenously inhibited by calpastatin [167]. Interestingly members of the Bcl-2 family are calpain substrates [168,169]; for example B-cell lymphoma-2 (Bcl-2) and Bcl-xL are both anti-apoptotic molecules but when cleaved by calpain they are converted to pro-apoptotic molecules [170]. On the other hand calpain cleavage of Bcl-2 associated protein X (Bax), a pro-apoptotic molecule, results in increased levels of its active form (a 18 kDa Bax) which possesses more potent cytotoxicity than uncleaved Bax [171]. A decreased ratio of Bcl-2or Bcl-xL to Bax favors cell death [172]. Gil-Parrado reported decreased formation of Bcl-2 and Bax heterodimer formation after calpain cleavage [173]. Caspases, also members of the cysteine protease family, are key executioners of apoptosis, with caspase-3 seen as the final killer in the apoptotic cascade [174]. The activation of caspase-3 can be mitochondrion-mediated due to the release of cytochrome c through opened mitochondrion permeable transition pores (MPTPs). Once in the cytosol, cytochrome c becomes associated with apoptotic protease activity factor-1 (Apaf-1), forming an apoptosome (a large complex molecule). Caspase-9, an initiator caspase becomes activated via protein-protein interaction with the apoptosome, which then activates the downstream effector caspase, caspase-3 [175]. It has been shown that taurine attenuates the amount of caspase-9 associated with Apaf-1 in ischemia [176]. This study provided evidence that taurine is protective at the Apaf-1/caspase-9 step of the mitochondrial-mediated apoptotic cascade. The translocation of cytosolic Bax to the mitochondrial membrane affects the MPTP; by forming a homodimer, Bax increases the opening of the MPTP. We and others [70] have shown that taurine attenuates mitochondrion-mediated-death pathways. Using a glutamate-induced neuronal damage culture system we have recently observed that taurine is able to shift the ratio of Bcl-2:Bax in favor of cell survival and that it also inhibits the glutamate-induced activation of calpain, resulting in an increased formation of Bcl-2/Bax heterodimers [70]. The increase in the formation of Bcl-2/Bax heterodimers cause a decrease in mitochondrial release of cytochrome c and an inhibition of the caspase-apoptotic cascade [70]. Protection against necrosis and apoptosis by taurine was also observed in experimental stoke models. In models of focal cerebral ischemia, taurine was reported to inhibit the ischemia-induced activation of m-calpain (no significant effect was observed on µ-calpain) in a dose-dependent manner by enhancing the expression and activity of calpastatin. Also in this model, Bax and caspase-3 were downregulated while Bcl2-xL was upregulated, which results in the attenuation of mitochondrial cytochrome c release and the consequential reduction of the mitochondrial-mediated apoptotic and necrotic cell death in the ischemic penumbra and core [177,178]. Taranukhin and colleagues also reported that taurine reduces ischemia-induced caspase-8 and caspase-9 expression (two upstream activators of caspase-3) in the paraventricular nucleus (PVN) and supraoptic nucleus (SON) of the rat’s hypothalamus [179]. 

### 3.3. Taurine’s Neuroprotective Effect against Endoplasmic Reticulum Stress

**Figure 2:**
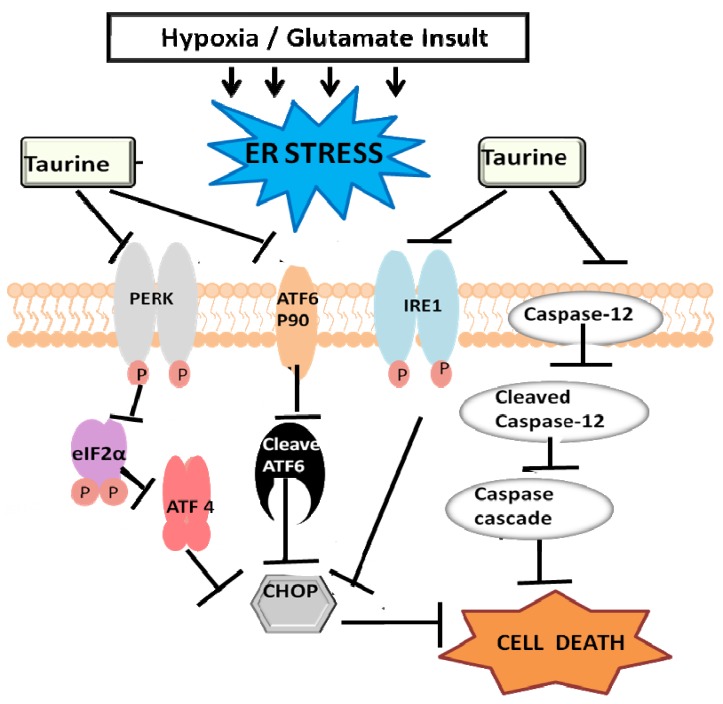
Schematic depiction of the ER-stress pathways and taurine’s neuroprotective effect on these pathways. During ER stress ATF6 (90 kDa) is cleaved to its active form, cleaved ATF6. Both PERK and IRE1 are also activated; by phosphorylation. Of the downstream players in the PERK pathway, phosphorylated eif2α activates the transcription factor ATF4 which translocates to the nucleus and transcribes CHOP. Cleaved ATF6 another transcription factor, also transcribes CHOP. Phosphorylated IRE1 along with its downstream players potentiates the expression of CHOP. CHOP, a transcription factor, transcribes pro-apoptotic proteins. Caspase-12, also localized at the ER membrane, is cleaved into its active form, cleaved caspase-12. Cleaved caspase-12 facilitates the caspase cascade apoptosis. Taurine inhibits the IRE1 and ATF6 pathways thereby attenuating cell death via ER stress [73,190].

In addition to the cell death mediated by the mitochondrion, increasing evidence points to ER stress as a critical player in hypoxic-ischemic cell death [109,180,181]. The ER is an essential sub-cellular organelle responsible for calcium storage and signaling, calcium-dependent processes such as the folding and processing of synthesized proteins and lipid biosynthesis [182,183,184]. Ischemic stroke induced ER stress, resulting in the impairment of ER protein folding [185]. An accumulation of unfolded/misfolded proteins activates the unfolded protein response (UPR) [186] mediated by ER transmembrane stress sensors, inositol-requiring kinase 1 (IRE1), double-stranded RNA-activated protein kinase 1 (PKR)-like endoplasmic reticulum kinase (PERK), and activating transcription factor 6 (ATF 6) [187]. Each stress sensor activates corresponding intracellular pathways (the IRE1-, PERK- and ATF6-pathways; Figure 2) that in turn mediate the up-regulation of the transcription factor C/EBP homologous protein (CHOP) also known as growth arrest and DNA damage inducible protein 153 (GADD 153) [188]. The UPR also activates caspase-12, an essential player in ER stress-mediated apoptosis [189]. In an *in vitro* model of hypoxia/reoxygenation, (0.3%, oxygen for 24 h, followed by reoxygenation at 21% oxygen for 24–48 h) we observed the neuroprotective effect of taurine against ER stress-mediated apoptosis [73]. In this study, taurine significantly increased the cell viability of the primary neuronal culture. The neuroprotective effect of taurine was dose-dependent, inhibiting the expression of CHOP and of caspase-12. The ratio of cleaved ATF6 to ATF6 declined by 50% in neurons treated with taurine relative to neurons exposed to hypoxia/reoxygenation alone, thereby inhibiting the ATF6-pathway. We also observed that taurine dramatically reduced the expression of p-IRE1 (the activated form of IRE1) in the IRE1 pathway but had no effect on the PERK pathway [73]. In a later study, using experimental stroke model of middle cerebral artery occlusion, (2 h ischemia followed by 4 days reperfusion) we observed that taurine attenuated infarct volume in 2 mm brain slices 6 mm from the frontal pole. Taurine’s neuroprotective effect on ER stress molecules was similar to our *in vitro* study; a reduction in the expression of CHOP, caspase-12, p-IRE1 and ATF6 [190]. We also observed that GRP78, another ER stress marker was reduced by taurine in this later study [190]. These studies provide convincing evidence that taurine is able to protect the ischemic brain against ER stress and subsequently ER stress mediated apoptosis, since CHOP; a transcription factor that upregulates the transcription of pro-apoptotic Bim (Bcl2 interacting mediator of cell death), and PUMA (p53 upregulated modulator of apoptosis) [191], while downregulating the transcription of anti-apoptotic Bcl2 [192], was downregulated by taurine. 

### 3.4. Taurine’s Neuroprotective Effect against Oxidative Stress, Inflammation and Edema

Other functions of taurine, such as anti-oxidative, anti-inflammatory or osmoregulatory could also contribute to its neuroprotective mechanism [19,72,193] against ischemic stroke. In the presence of high mitochondrial calcium, the physiological production of reactive oxygen species [(ROS: such as superoxide anion (O_2_^−^), hydrogen peroxide (H_2_O_2_) and hydroxyl radical (·OH)] and nitric oxide NO, becomes pathological due to an imbalance of their production versus their degradation [194]. This pathological condition is commonly referred to as oxidative stress and it is observed in ischemic stroke [195]. The excessive intra-mitochondrial calcium [Ca]_m_ is able to generate excessive ROS by one or all of such methods. Firstly, [Ca]_m_ stimulates the tricarboxylic Acid (TCA) cycle, which enhances electron flow into the electron transport respiratory chain, increasing the mitochondrion’s work and simultaneously increase the respiratory chain electron leakage to the acceptor O_2_, generating ROS [196,197]. Secondly, [Ca]_m_ disrupts the electron transport respiratory chain by either affecting conformational changes [198] in the respiratory complexes, exemplified by changes in complex lV (Wikstrom and Saari [199] or by activating the Ca^2+^-dependent production of nitric oxide (via nitric oxide synthase), which inhibits complex I directly [200] and complex III in conjunction with calcium [201] of the respiratory chain. The release of cytochrome *c* from the mitochondria inhibits complex lV [202]. This bottle neck in the respiratory chain diverts the flow of electrons from the chain to O_2_. Thirdly, [Ca]_m_ inactivates ROS scavengers, such as glutathione peroxidase (GPx) [203] resulting in a decrease in antioxidant capacity in the mitochondria. It is to be noted as well that the loss of GPx via an opened MPTP further reduces mitochondrial GPx [204]. Recci and colleagues [205] attributed the loss of mitochondrial respiratory chain integrity to a decline in the synthesis of the encoded proteins in the respiratory complexes.

Several lines of evidence have shown taurine to be protective against oxidative injury [65,206,207,208,209,210]. Taurine reduces ROS not by directly scavenging ROS [211] but instead by potentiating or rescuing endogenous anti-oxidants, as reported by many of these studies. Interestingly, Jong and colleagues reported that taurine’s anti-oxidative effect is due to the maintenance of the mitochondrial respiratory chain integrity by taurine [212]. By using β-alanine, an inhibitor of taurine-linked reactions [213] they showed a reduction in complex l and complex III activity of the mitochondrial respiratory chain with a simultaneous reduction in oxygen consumption and an increase in mitochondrial oxidative stress (enhanced superoxide production, oxidation of glutathione and inactivation of aconitase, an oxidant sensitive enzyme). The reduction in complex 1 activity correlates with a reduction in the synthesis of mitochondrial proteins, ND5 and ND6; proteins that are apart of complex 1 protein assembly and are also encoded by taurine-conjugated mt tRNA^Leu(UUR)^. The encoding function of mt tRNA^Leu(UUR)^ was impaired by the taurine competitor, β-alanine. The reductions of oxidants reduce cellular damage such as membrane lipid peroxidation.

An inflammatory reaction occurs in response to brain ischemic stroke due to the infiltration of neutrophils, macrophages, activated microglia and inflammatory mediators such as various cytokines, adhesion molecules, and chemokines [214]. The transcription factor, nuclear factor-κB (NF-κB), enhances the production of inflammatory mediators by transcribing inflammatory genes. NF-κB’s action is potentiated by poly-ADP-ribose polymerase (PARP) which is reported to act as its co-activator [215,216]. Inflammation was reduced by taurine (50 mg/kg b.wt.) in a rat model of transient focal ischemia [217]. These investigators observed that the up-regulation of PARP and NF-κB in the ischemic core and penumbra was reversed by taurine and that the levels of the inflammatory cytokines, tumor necrosis factor-α and interleukin-1β, were significantly reduced [217]. Edema is one of the resulting conditions after a brain ischemic insult. This is caused by an accumulation of intracellular Na^+^ and Cl^−^ which instigates osmotic water influx [218]. Swelling then potentiates taurine’s release; initially release is exocytosis and Ca^2**+**^-dependent, subsequently through the reverse mode of the Na^+^/Cl^−^-dependent TauT [219,220], as the ischemic insult prolong, release is through volume-sensitive chloride channels [221,222,223,224], and finally by diffusing across a permeabilized plasma membrane [99]. This regulates the cell’s volume, preventing cell death by necrotic swelling. It was shown that taurine significantly reduced cell swelling in rat brain cortical slices after exposure to oxygen-glucose deprivation and reoxygenation [141]. 

In spite of the plethora of convincing demonstrations of the neuroprotective effect of taurine in ischemic stroke there are conflicting reports showing the failure of taurine to protect against this type of brain insult. Shuaib, reported that taurine (100 mg/kg, i.p.) did not statistically reduce infarct volume [225]. This contradictory report could be due to differences in experimental conditions, animal model, and route of administration or more interestingly, by a dose-dependent biphasic response of taurine in which a low concentration of taurine (1 mM) elicits hyperpolarization but at a higher concentration (10 mM), hyperpolarization is followed by slow depolarization [226]. Taurine’s biphasic effect in an ischemic insult was clearly seen in an *in vivo* model of hypoxia-induced convulsion where taurine suppresses convulsion in a dose-dependent manner but at a high dose of 100 mg/kg no protection was observed [227]. We have also demonstrated the biphasic effect of taurine in excitatory amino acid-induced neurotoxicity in primary neuronal cultures [136]. The biphasic response of taurine cautions investigators about the dose to be administered in their experiments which should be empirically determined, especially when using different experimental models. 

## 4. Clinical Trial

There is a growing body of preclinical data that demonstrates taurine’s neuroprotective effect in cerebral ischemic stroke. It is a lipophobic amino acid and while the BBB prevents significant amount of exogenous taurine from entering the brain, in cerebral ischemia the BBB is damaged [228] which allows free access of exogenous taurine to injured neurons and glia. Inspite of accumulating data there seems to be a paucity of studies on taurine in clinical trials for stroke. Much of the human trials with taurine involving ischemia-reperfusion injury were reported for cardiovascular diseases [229,230,231,232,233]. For instance, Azuma and colleagues orally administered taurine (3 mg/day) to 17 patients with congestive heart failure (CHF: observed as reduced left ventricular function), a secondary condition of heart ischemia, for 6 weeks. They reported that there was a significant improvement in systolic left ventricular function in the taurine-treated group compared to the group treated with coenzyme Q10 [230]. Similar effect of taurine to improve congestive heart failure was observed in an earlier clinical study by Azuma and colleagues [229]. In this study the group given taurine orally showed significant improvement in CHF over the group given placebo. No adverse effect from taurine was reported in either study. Interestingly other investigators provided evidence that an intake of dietary taurine improved ischemic heart disease. Yamori and colleagues performed an epidemiological study (in 19 centers of 14 countries, including both sexes) of taurine’s effect on ischemic heart disease. They used 24 h urinary (24-U) taurine excretion as a biological marker for dietary intake of taurine. They reported that there was a significant inverse correlation with the 24-U taurine excretion and ischemic heart disease in both sexes [231]. Later Yamori and colleague conducted another but similar epidemiology study using a larger population [232]. The findings from the later study confirmed that of their former study. We postulate that the high intake of dietary taurine could be protecting the heart cells from further death due to ischemia in these epidemiology studies by Yamori.

Recently Rikimaru and colleagues reported that the addition of taurine to the culture media of MELAS patient-derived cells ameliorated reduced oxygen consumption, improves the mitochondrial membrane potential and reduced oxidative stress [160]. In the same study Rikimaru and colleagues reported that taurine protected the brains of two MELAS patients from the spread of stroke-like lesions, revealed by MRI. Taurine neuroprotective effects in these patients were manifested in an amelioration and eventually complete cessation of stroke-like episodes. In this small clinical study a daily dose of taurine (0.25 g/kg/day) was administered for over a period of nine years but protection was observed from the beginning of the study (year one) as seen from MRI of the brain and the alleviation of symptomatic stroke-like episodes [160]. Apart from this small study there seems to be no other clinical study on taurine in stroke patients. The deficiency of taurine usage in cerebral ischemic stroke trials could be due to insufficient preclinical experiments that have rigorously validated the Stroke Therapy Academic Industry Roundtable (STAIR) criteria for neuroprotective stroke agents. An updated version of the STAIR criteria includes: (1) Identification of the minimum effective and maximum tolerable dosage. (2) Identification of a therapeutic window; suggested usage of mismatched between perfusion-weighted MRI (monitors blood supply of the tissue) and diffusion-weighted MRI (measures tissue damage) of the penumbra would be a useful aid for this identification. (3) Both histological and behavioral measurements of experimental studies should be endpoint assessments and that studies should be performed over a 2–3 week after stroke on-set, to demonstrate sustainability of the tested compound. (4) Physiological parameters such as blood pressure, body temperature, blood gases and glucose should be routinely monitored. A Doppler Flow apparatus or perfusion MRI should be used to monitor decreased blood flow and reperfusion in temporary ischemic models. (5) Data obtained in one laboratory should be replicable in at least one other independent laboratory. (6) Efficacy study should be done on animals of both sexes and of all ages, interaction between tested compound and medication commonly used by stroke patients should be performed, serum markers of tissue injury similar to those obtainable in human trial should be used and studies should include more than one type of animal species. In addition, studies in animals with comorbidities such as hypertension, diabetes and hypercholesterolemia should be carried out if this is the targeted population for human trial [234]. For a successful translation to clinical trials more preclinical experiments need to be performed with the STAIR’s criteria in mind. Similarly, more clinical studies need to be done on the changes of taurine levels in stroke patients which would provide insight as to whether changes caused by ischemic stroke are the same in animal and patients [235]. This would provide a more credible extrapolation from experimental studies to patients.

Another concern in translating taurine from the bench to the bedside is the potential of any adverse side effects. Taurine is a naturally occurring amino acid of the body and should therefore exhibit minimal, if any, adverse side effects. Toxicology studies have not reported any genotoxic, carcinogenic or teratogenic effects of taurine [236,237,238,239]. An area of controversy on the safety issue of taurine usage is in the maximum tolerable dosage of taurine/No Observed Adverse Effect Level (NOAEL). Furukawa and colleagues reported that a 13 week intravenous taurine treatment resulted in water consumption (1000 and 2000 mg/kg b.wt/day taurine intake) and haemosiderin (a denatured ferritin complex which poorly provide available iron when needed) deposition in the lungs (2000 mg/kg b.wt./day taurine intake) [240]. The authors concluded that the NOAEL was 500 mg/kg b.wt./day [240]. While Cantafora and others reported that taurine (462 mg/kg b.wt./day) administered in drinking water of guinea pigs for two weeks resulted in fatty infiltration of the liver [241]. Recently the European Food Safety Authority (EFSA) shared their opinion that 1000 mg/kg b.wt./day was the NOAEL for taurine in energy drinks [242]. Attention should be drawn to the fact that this dose (1000 mg/kg b.wt./day) of taurine was in energy drinks which consisted of other active compound and therefore any potential adverse effect at this dose of taurine maybe camouflaged by the other ingredients. This dosage (1000 mg/kg b.wt.) may also not be neuroprotective in cerebral ischemia due to taurine’s biphasic mechanistic mode of action (commented on previously) observed in ischemic preclinical studies [136,225,226,227].

## 5. Concluding Remarks

Taurine is synthesized within brain cells and exhibits a plethora of physiological functions [19]. It has a multiple of neuroprotective mechanisms in the CNS such as: regulating cellular osmolarity [243,244], an anti-oxidant [65,66], neuromodulator of GABAergic transmission [85,245,246], maintaining calcium homeostasis [59,60,61,62,63], inhibiting glutamate excitotoxicity [59,69,133], attenuating endoplasmic reticulum stress [73,190,247], modulating the mitochondrial pore permeability [155] downregulating a range of pro-apoptotic proteins while upregulating anti-apoptotic proteins [70,176,179,248] and downregulating inflammatory mediators [217]. In ischemic stroke, a pathological brain condition, taurine is released in the extracellular space resulting in a decrease in the concentration of intracellular taurine. The decrease in intracellular: extracellular taurine ratio attenuates the protective role of taurine and could potentiate neuronal damage during ischemia. The administration of exogenous taurine protects the neuropile in ischemic stroke, evident from numerous experimental reports [23,63,65,69,70,73,140,141,142,144,154,155,156,160,176,179,190,206,207,208,209,210,212,217]. Exogenous taurine is able to elicit its neuroprotective mechanisms both at the intracellular and the extracellular level. Taurine administered subcutaneously (s.c.), intravenously (i.v.) or intraperitonely (i.p.) is able to cross the BBB in ischemia [228]. Once it crosses the BBB it may be transported into cells via the taurine transporter (TauT) [249,250], where it is able to mediate its protective mechanisms in key subcellular organelles suchthe mitochondria and the endoplasmic rectiulum. In the mitochondrion, taurine buffers [Ca]_m_ [63] and the mitochondrial pH [154,156], two important parameters that maintain the integrity of the mitochondrial membrane potential (Δψm), preventing mitochondrial-mediated apoptosis via the activation of caspases [70,155,178]. Within the mitochondrion, taurine may also attenuates excessive ROS generated in ischemic stroke [65,212]. Taurine protects the endoplasmic reticulum from being stress, evidential by the reduction of ER stress markers such as CHOP and caspase-12 in taurine-treated experimental models [73,190,247].

**Figure 3:**
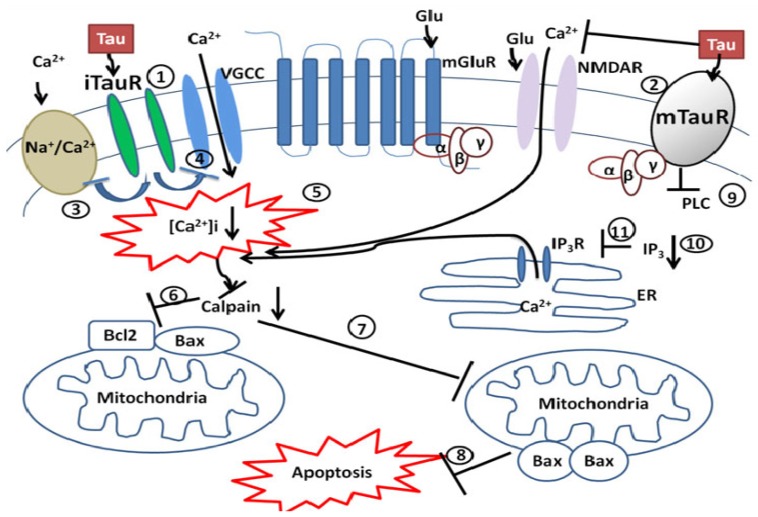
Schematic depiction of taurine neuroprotective mechanisms via putative taurine receptors. (**1**) Activated ionotropic taurine receptor (iTauR) and/or (**2**) metabotropic taurine receptor (mTauR) inhibits (**3**) the reverse mode of the sodium/calcium exchanger; (**4**) inhibition of voltage-gated calcium channels (VGCC) due to taurine induced hyperpolarization, decreases (**5**) intracellular calcium. Reduction in intracellular calcium inhibits calpain, eliciting the inhibition of (**6**) calpain-induced cleavage of Bcl-2 and Bax. (**7**) Bax homodimer is inhibited, resulting in the inhibition of the (**8**) mitochondrion-mediated death cascade. (**9**) Phospholipase C (PLC) is inhibited by activated mTauR (mTauR: is coupled to inhibitory G-protein), resulting in (**10**) decreased IP_3_ production, which attenuates (**11**) the release of calcium from the endoplasmic reticulum(ER) causing a reduction of ER stress and ER stress-mediated apoptosis [49].

Several lines of evidences have reported that extracellular taurine modulates inhibitory neurotransmission via GABA_A_ and glycine receptors [83,85,143]. The activation of these inhibitory receptors attenuates the influx of calcium, protecting the ischemic brain against glutamate-mediated apoptosis [144]. We have also demonstrated that taurine counteracts the glutamate-induced increase of intracellular calcium through L-, P/Q-, N-type voltage-gated calcium channels (VGCCs) and the *N*-methyl-d-aspartate (NMDA) receptor, thus preventing glutamate induced membrane depolarization [59,69]. Although there is no cloned taurine receptor, several studies have provided strong evidence of the existence of a specific taurine receptor [86,251,252,253,254]. In our previous studies [86], we demonstrated that the receptor is neither activated nor antagonized by structurally similar amino acids such as glutamate, GABA and glycine. These observations were later supported by Frosini and colleagues [87]. There is also a strong possibility of there being two types of taurine receptor; an ionotropic taurine receptor [88] and a metabotropic taurine receptor [60]. Other researchers have also demonstrated the existence of distinct types of taurine receptor [226]. We propose that taurine’s neuroprotective effect against glutamate-induced apoptosis is in part mediated via these receptors (Figure 3).

The possibility of a metabotropic taurine receptor that is coupled to an inhibitory G-protein (Foos and Wu 2002 [60]) resulting in reduction of ER calcium release, maintaining the ER’s calcium homeostasis in the ischemic brain (Figure 3), could provide insights into taurine’s mechanism in reducing ER stress and ER stress-mediated apoptosis [73,190]. The neuroprotective mechanisms of taurine address the diverse pathological mechanisms observed in ischemic stroke. Taurine not only addresses stroke pathology but it also extends the therapeutic window in which a compound maybe effective. Sun and colleagues, using an experimental rat model of stroke demonstrated that intravenous administration of taurine (50 mg/kg) was neuroprotective up to 8 h after ischemia [217]. They attributed the suppression of neutrophil infiltration as one of the neuroprotective mechanisms of delayed taurine administration. Several preclinical studies have provided substantiating evidence of taurine’s neuroprotective mechanism against cerebral ischemia but for taurine to successfully translate to clinical trials of stroke patients, more preclinical research needs to be carried out that rigorously meets the STAIR criteria for a neuroprotective agent in cerebral stroke research [234]. On the other hand, taurine has had success in clinical trials on congestive heart failure (CHF) [229,230,231,232,233] where the specific dosage and length of administration did not produce any adverse side effects. This should alert clinical stroke researchers to the feasibility of applying the CHF clinical paradigm to cerebral stroke patients. 
